# Cycloartane triterpenoid (23*R*, 24*E*)-23-acetoxymangiferonic acid inhibited proliferation and migration in B16-F10 melanoma via MITF downregulation caused by inhibition of both β-catenin and c-Raf–MEK1–ERK signaling axis

**DOI:** 10.1007/s11418-018-1233-7

**Published:** 2018-08-06

**Authors:** Toshio Kaneda, Misaki Matsumoto, Yayoi Sotozono, Satoshi Fukami, Alfarius Eko Nugroho, Yusuke Hirasawa, Hadi A. Hamid A, Hiroshi Morita

**Affiliations:** 1 Faculty of Pharmaceutical Sciences, Hoshi University, Ebara 2-4-41 Shinagawa-ku, Tokyo, 142-8501 Japan; 2 Department of Chemistry, Faculty of Science, University Malaya, 50603 Kuala Lumpur, Malaysia

**Keywords:** *Garcinia*, Triterpenoids, Cycloartane, Melanoma, MITF, β-Catenin, ERK, c-Raf

## Abstract

We recently reported that (23*R*, 24*E*)-23-acetoxymangiferonic acid (23*R*-AMA), a cycloartane triterpenoid isolated by activity-guided separation from a methanol extract of *Garcinia* sp. bark, inhibited melanin production via inhibition of tyrosinase (TYR) expression in the B16-F10 melanoma cell line. Since 23*R*-AMA also inhibited microphthalmia-associated transcription factor (MITF) expression, an upstream factor of TYR, these features of 23*R*-AMA were thought to be appropriate for development of whitening cosmetics. However, 23*R*-AMA exhibited growth inhibition other than inhibition of melanin production in B16-F10 cells. Therefore, we investigated biological activities of 23*R*-AMA in detail, focused on its application as an anti-melanoma compound. In this study, we demonstrated that 23*R*-AMA inhibited cell proliferation and basic FGF (bFGF)-induced migration in B16-F10 cells. Furthermore, 23*R*-AMA promoted ser45/thr41 phosphorylation of β-catenin and suppressed its intranuclear accumulation, which was suggested to be related to inhibition of MITF expression. The transcriptional activity of MITF is known to be regulated by phosphorylation via activated ERK. Further investigation revealed that 23*R*-AMA inhibited phosphorylation of c-Raf, MEK-1, and ERK, and also that of upstream molecules including FAK and c-Src. These results suggested that 23*R*-AMA inhibited growth and migration of B16-F10 melanoma by regulating both MITF expression and its activity. The activities of 23*R*-AMA reported in this study are new aspects of cycloartane triterpenoids.

**Electronic supplementary material:**

The online version of this article (10.1007/s11418-018-1233-7) contains supplementary material, which is available to authorized users.

## Introduction

Melanocytes are cells producing melanin pigments in the basal layer under the epidermis. Melanin formation starts from hydroxylation of l-tyrosine to l-DOPA, which is the rate-limiting step in melanin synthesis and is catalyzed by tyrosinase (TYR) located in melanosomes. Melanin pigments prevent cell damage from ultraviolet rays by covering the nucleus. Since excessive accumulation of melanin causes blemishes and freckles, a lot of studies have sought a component having whitening effects such as inhibiting melanin generation and/or promoting melanin decomposition. In the process of melanin synthesis in stimulated melanocytes, such as by ultraviolet rays and friction, microphthalmia-associated transcription factor (MITF) is a master transcription factor, which promotes gene expression of TYR, tyrosinase-related protein-1 (TRP-1), and TRP-2. Mature melanosomes are transported to the keratinocytes by dendrites, and are moved to the skin surface with the differentiation of the keratinocytes [1]. The melanocytes produce melanin to protect somatic cells from ultraviolet rays, but these cells may be transformed to malignant melanoma by oncogenesis.

Melanoma is a type of skin cancer whose worldwide incidence has steadily increased over the last several decades. Annual incidence has risen as rapidly as 4–6% in many fair-skinned populations that predominate in regions like North America, Northern Europe, Australia, and New Zealand [2]. Melanoma is known as the most malignant skin cancer with a high fatality rate since its progressing state shows resistance to various treatments. It has been reported that MITF expression is elevated or mutated in melanoma [3]. Recently, MITF-M, one of the isoforms of MITF, was reported to be specifically expressed in melanoma cells [4]. Furthermore, the forced expression of MITF caused tumorigenesis of immortalized melanocytes. Apoptosis of malignant melanoma was induced by functional inhibition of MITF [5]. These reports indicated that MITF is a pathogenic factor in melanoma and a potential target molecule for therapy.

We recently reported that (23*R*, 24*E*)-23-acetoxymangiferonic acid [(23*R*, 24*E*)-23-acetoxy-3-oxocycloart-24-en-26-oic acid] (23*R*-AMA), a cycloartane triterpenoid isolated from a methanol extract of *Garcinia* sp. bark, has inhibitory activity against melanin production via inhibition of TYR expression in the B16-F10 melanoma cell line [6]. Plants of the genus *Garcinia* are evergreen trees of the Clusiaceae family. Xanthones, such as α-mangostin and gambogic acid, and (−)-hydroxycitric acid have been isolated from plants of the genus *Garcinia*, and these compounds have been reported to have anti-inflammatory, antioxidant, and antitumor activity [7–10]. Cycloartane triterpenoids, such as euphonerin D [11] isolated from *Euphorbia neriifolia,* and combretic acid B and combretanone G [12] isolated from *Combretum quadrangulare*, have been reported to show an antitumor effect by apoptosis induction by increasing DR5 promoter activity. In addition, it has been reported that seven kinds of cycloartane-type triterpenoids, including cycloartenol, isolated from *Amberboa ramosa* have TYR inhibitory activity [13]. Thus, numerous compounds having a cycloartane skeleton with various bioactivities have been reported. However, no detailed mechanism of action has been investigated for cycloartane-type triterpenoids isolated from plants of the genus *Garcinia*.

In this study, we investigated the detailed mode of action of 23*R*-AMA-induced inhibitory effects on cell proliferation and migration in B16-F10 melanoma, and found that these activities were caused by inhibitory regulation to both MITF expression and its transcriptional activity, and which were elicited by inhibition of β-catenin and c-Raf–MEK1–ERK signaling axis including FAK and c-Src.

## Materials and methods

### Materials

The barks of *Garcinia *sp. were collected in Johor, Malaysia in August 2003. The botanical identification was made by Mr. Teo Leong Eng, Faculty of Science, University of Malaya. Voucher specimens (Herbarium No. 5044) are deposited in the Herbarium of the Chemistry Department, University of Malaya. Details of the structure elucidation of 23*R*-AMA extracted from this plant were described in a recent report [6].

1-*tert*-Butyl-3-(4-chlorophenyl)-1*H*-pyrazolo[3,4-*d*]pyrimidin-4-amine (PP2), an Src family kinase inhibitor, was purchased from Abcam (Cambridge, UK).

### Cell culture

The B16-F10 melanoma cell line was provided by ATCC (Manassas, VA, USA) and cultivated in RPMI 1640 medium (Wako Pure Chemical Industries, Ltd., Osaka) containing 10% FBS (Equitec-Bio, Inc., TX, USA) and penicillin–streptomycin (Wako Pure Chemical Industries, Ltd.).

### Drug evaluation by melanin production

B16-F10 cells were seeded at a density of 5.0 × 10^4^ cells/well in a 24-well plate, and 3-isobutyl-1-methylxanthine (IBMX; Wako Pure Chemical Industries, 100 μM) and [Nle^4^, d-Phe^7^]-α-melanocyte-stimulating hormone trifluoroacetate salt (α-MSH; Sigma-Aldrich, St. Louis, MO, USA, 0.25 μM) as a melanin production inducer, various concentrations of 23*R*-AMA (6.25–25 μg/mL; 11.7–46.7 μM), and arbutin (750 μM) as a positive control were added, and the cells were cultured for 72 h. Cells were lysed with 0.1 M NaOH and total melanin content was measured by absorbance at 360 nm. In addition, the protein content of the cell lysate was measured with Coomassie Protein Assay Reagent (Thermo Scientific, Waltham, MA, USA) by absorbance at 595 nm.

### Evaluation of cytotoxic effects by lactate dehydrogenase (LDH) activity and cell proliferation

B16-F10 cells were seeded in a 96-well plate at a density of 1.0 × 10^4^ cells/well and various concentrations of 23*R*-AMA (3.13–50 μg/mL; 5.8–93.4 μM) were added. After cultivation for 24 h, LDH activity of the culture medium was evaluated by Cytotoxicity LDH Assay Kit-WST (Dojindo, Kumamoto) according to the instruction manual. B16-F10 cells cultured under the same conditions as the LDH assay were collected by trypsin treatment and counted with a hemocytometer.

### Migration assay

B16-F10 cells were seeded in a chemotaxis chamber (pore size 3 μm; BD Biosciences, Franklin Lakes, NJ, USA) set on 24-well plates. After 12 h cultivation with/without 23*R*-AMA (12.5 μg/mL; 23.4 μM), basic FGF (bFGF: Wako Pure Chemical Industries, Ltd.) was added in the lower compartment, and cells were cultivated for 24 h. The cells that moved through to underneath the membrane were washed with ice-cold PBS, fixed with 10% formalin, and stained with 3% Giemsa solution (MERCK KGaA, Darmstadt, Germany). For quantification of migrated cells, the cells remaining on the membrane of the upper layer were all removed with a cotton swab, and MTT assay was performed on the migrating cells remaining under the membrane. Formazan produced by adding MTT solution to the lower layer was dissolved with DMSO and its absorbance at 570 nm was measured [14].

### Flow cytometry analysis

Cells subjected to various treatments were washed with ice-cold PBS and then washed with assay buffer [10 mM Hepes (pH 7.4), 137 mM NaCl, 1 mg/mL glucose, 0.5 mM EDTA, 0.001% NaN_3_, 0.3% BSA] and collected. After cells were stained with various antibodies, the fluorescence of FITC and PE on cells was detected with a flow cytometer (FACSVerse; BD Biosciences). When the biotinylated antibody was used, cells were stained with streptavidin APC-Cy™7 (BD Pharmingen, Franklin Lakes, NJ, USA) after the primary antibody binding, and its fluorescence was analyzed.

### Reverse Transcription–Polymerase Chain Reaction (RT-PCR)

Total RNA (2 μg) extracted from cells in the culture was used as a template for cDNA synthesis. cDNA was prepared by use of a Rever Tra Ace (TOYOBO Co., Ltd., Osaka). Primers were synthesized on the basis of the reported mouse mRNA sequences for GAPDH, MITF, TYR, c-MET, TCF1, integrin α_V_, integrin α_4_, integrin α_5_, integrin β_1_, and integrin β_3_. Sequences of the primers used for PCR were as follows. GAPDH: forward 5′-TCATCATCTCCGCCCCTTC-3′, reverse 5′-TGCCTGCTTCACCACCTTCT-3′; MITF: forward 5′-GTGCAGACCCACCTGGAAAAC-3′, reverse 5′-AGTTAAGAGTGAGCATAGCCATAG-3′; TYR: forward 5′-GATCAGAAGAGTATAATAGCCAT-3′, reverse 5′-CAATATAAGGGCTGTAAAAGCCT-3′; c-MET: forward 5′-ATTGGACCCAGCAGCCTGATTG-3′, reverse 5′-AGCTTGGCACCCGTCTTGTTGT-3′; TCF1: forward 5′-CCAACATTCTCAGGTCGCTCT-3′, reverse 5′-GGCCTAAACGTATCCTAGTCCC-3′; integrin α_V_: forward 5′-GCCAGCCCATTGGAGTTTGATT-3′, reverse 5′-GCTACCAGGACCACCGAGAAG-3′; integrin α_4_: forward 5′-GGCTCCATCATCAAAGACC-3′, reverse 5′-AAATCATTCCCTTTAAGTCGG-3′; integrin α_5_: forward 5′-AATGCCCTGAAGCCAAGTGTT-3′, reverse 5′-TCAGGAAGTGCCTAGTCCCT-3′; integrin β_1_: forward 5′-TGGCAACAATGAAGCTATCGT-3′, reverse 5′-GTGTTGCAAAATCCGCCTGA-3′; integrin β_3_: forward 5′-CCTCTTCGGCTACTCGGTC-3′, reverse 5′-ACTCCAAGCCACATCTCCTCA-3′.

Amplification was conducted for 22–32 cycles, each of 94 °C for 30 s, 58 °C for 30 s, and 72 °C for 1 min in a 25 μL reaction mixture containing 25 ng/5 μL of each cDNA, 25 pmol of each primer, 0.2 mM dNTP, and 1 U of *Taq* DNA polymerase (Qiagen, Valencia, CA, USA). After amplification, 15 μL of each reaction mixture was analyzed by 1.5% agarose gel electrophoresis, and the bands were then visualized by ethidium bromide staining.

### Western blot analysis

After 23*R*-AMA and IBMX treatments, the cells were washed with PBS, harvested with 2 × Laemmli buffer [125 mM Tris–HCl (pH 6.8), 4% SDS, 20% glycerol, 0.0025% bromophenol blue (BPB)], and then sonicated for 15 s. The protein concentration in the cell lysate was measured with a bicinchoninic acid protein assay kit (Thermo scientific). Each sample containing equal amounts of protein was subjected to 7–10% SDS–polyacrylamide gel electrophoresis (PAGE), and the proteins separated in the gel were subsequently electrotransferred onto a polyvinylidene difluoride membrane. After being blocked with 5% skim milk, the membrane was incubated with goat anti-TYR antibody (Santa Cruz Biotechnology, Santa Cruz, CA, USA), mouse anti-MITF antibody (Thermo scientific), mouse anti-Bax, anti-Bcl-2, anti-Bcl-xL, anti-Fyn(pTyr528)/c-Src(pTyr530), rabbit anti-Mcl-1 (BD Biosciences), mouse anti-MEK1/2, rabbit anti-c-Raf, anti-LC3, anti-Bad, anti-Bid, anti-mTOR, anti-β-catenin, anti-p-β-catenin (Ser33/Ser37/Thr41, Ser45/Thr41, Ser552, Ser675), anti-ERK1/2, anti-p-ERK1/2, anti-p-MEK1/2, anti-p-c-Raf, anti-B-Raf, anti-p-B-Raf antibody (Cell Signaling Technology, Inc., Beverly, MA, USA) and subsequently with peroxidase-conjugated anti-mouse, anti-rabbit IgG antibody (GE Healthcare, Fairfield, CT, USA) or anti-goat antibody (Santa Cruz Biotechnology). Immunoreactive proteins were visualized with western blot chemiluminescence reagents (Wako Pure Chemical Industries, Ltd.).

For preparation of nuclear protein extract, cells subjected to various treatments were washed with PBS, and hypotonic buffer [10 mM Hepes (pH 7.4), 10 mM KCl, 0.1 mM EDTA, 0.1% NP-40, 1 mM DTT (dithiothreitol), 1 mM 4-(2-aminoethyl)benzensulfonyl fluoride hydrochloride (ABSF), 2 μg/mL aprotinin, 2 μg/mL pepstatin, 2 μg/mL leupeptin] was added. The collected solution was centrifuged at 2400 × *g* at 4 °C for 5 min, the supernatant was removed. Hypertonic buffer [50 mM Hepes (pH 7.4), 420 mM KCl, 0.1 mM EDTA, 5 mM MgCl_2_, 20% glycerol, 1 mM DTT, 1 mM ABSF, 2 μg/mL aprotinin, 2 μg/mL pepstatin, 2 μg/mL leupeptin] was added to precipitate, and the mixture was stirred by tapping for 1 h on ice. Centrifuged at 13,800 × *g*, 4 °C for 15 min, and the supernatant was used as a nuclear extracted protein. Coomassie Protein Assay Reagent (Thermo scientific) was used for protein quantification and diluted with hypertonic buffer to contain equal amounts of protein [15].

## Results

### 23*R*-AMA-induced inhibition of melanin production

**Fig. 1:**
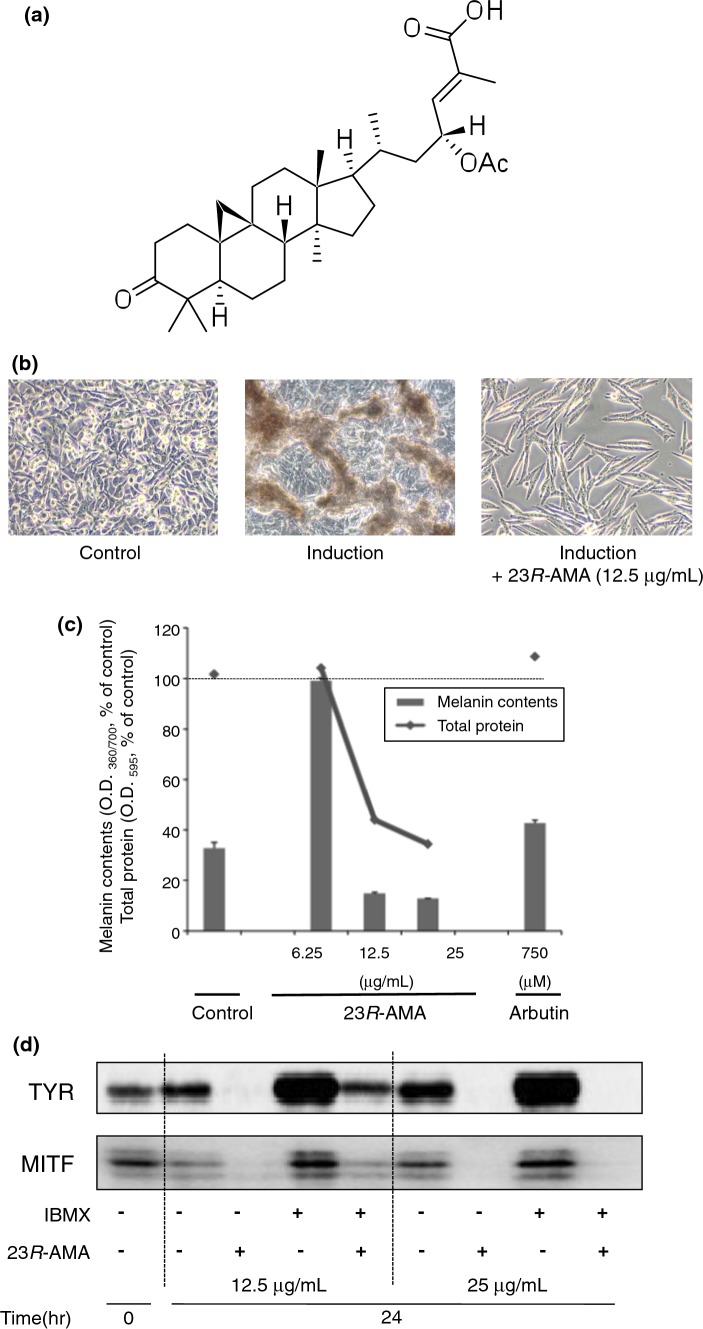
23*R*-AMA-induced inhibition of melanin production. **a** Structure of 23*R*-AMA [6]. **b** B16-F10 cells were seeded in a 24-well plate at 5.0 × 10^4^ cells/well. α-MSH (0.25 μM) and IBMX (100 μM) as melanin inducer and 23*R*-AMA (12.5 μg/mL) were added and cultured for 72 h. **c** B16-F10 cells were seeded in a 24-well plate at 5.0 × 10^4^ cells/well, and IBMX (100 μM), α-MSH (0.25 μM), and various concentrations of 23*R*-AMA (6.25, 12.5, 25 μg/mL) were added and cultured for 48 h. Cells were lysed with 50 μL of 0.1 M NaOH and the total melanin content and protein concentration were measured by absorbance at 360 and 595 nm, respectively. Arbutin (750 μM) was applied as a positive control. **d** B16-F10 cells were seeded in a 60-mm dish at 6.0 × 10^5^ cells/dish, and then IBMX (100 μM) was added as a melanin production inducer. IBMX-stimulated cells with/without 23*R*-AMA (12.5, 25 μg/mL) were harvested after 24 h cultivation, and western blot was performed using specific antibodies to TYR [6] and MITF

The addition of α-MSH and IBMX promoted melanin production in 3-day cultivation. Addition of 23*R*-AMA (Fig. 1a) completely suppressed melanin accumulation in B16-F10 with morphological change (Fig. 1b). 23*R*-AMA strongly inhibited melanin content to 15% at 12.5 μg/mL and to 12% at 25 μg/mL, as compared with the induced group that was stimulated by α-MSH and IBMX (Fig. 1c, 100%). Melanin content in the 23*R*-AMA-treated group was less than in the non-treated group without α-MSH and IBMX. The positive control arbutin (750 μM), which is a versatile whitening agent, suppressed melanin content to 43% (Fig. 1c). Such inhibition by 23*R*-AMA shown in Fig. 1b, c was also observed in melanin induction by only IBMX. Therefore, only IBMX was used as melanin inducer in the following experiments to simplify analyses.

The protein expression of TYR and MITF, a key enzyme and a transcription factor, respectively, in B16-F10 treated with 23*R*-AMA was examined by western blotting (WB). Protein expression of TYR and MITF is increased by 24 h after IBMX addition, but these expressions were completely suppressed by addition of 23*R*-AMA (Fig. 1d). Furthermore, 23*R*-AMA-induced reduction of protein expression of both TYR and MITF was observed in the samples without IBMX. In these experimental conditions, although 23*R*-AMA-inhibited cell proliferation with morphological change (Fig. 1b, c) was obvious, noticeable cytotoxic effects were not observed (Fig. 1b).

## Inhibition of cell proliferation by 23*R*-AMA and influence on apoptosis- and autophagy-related protein expression

**Fig. 2:**
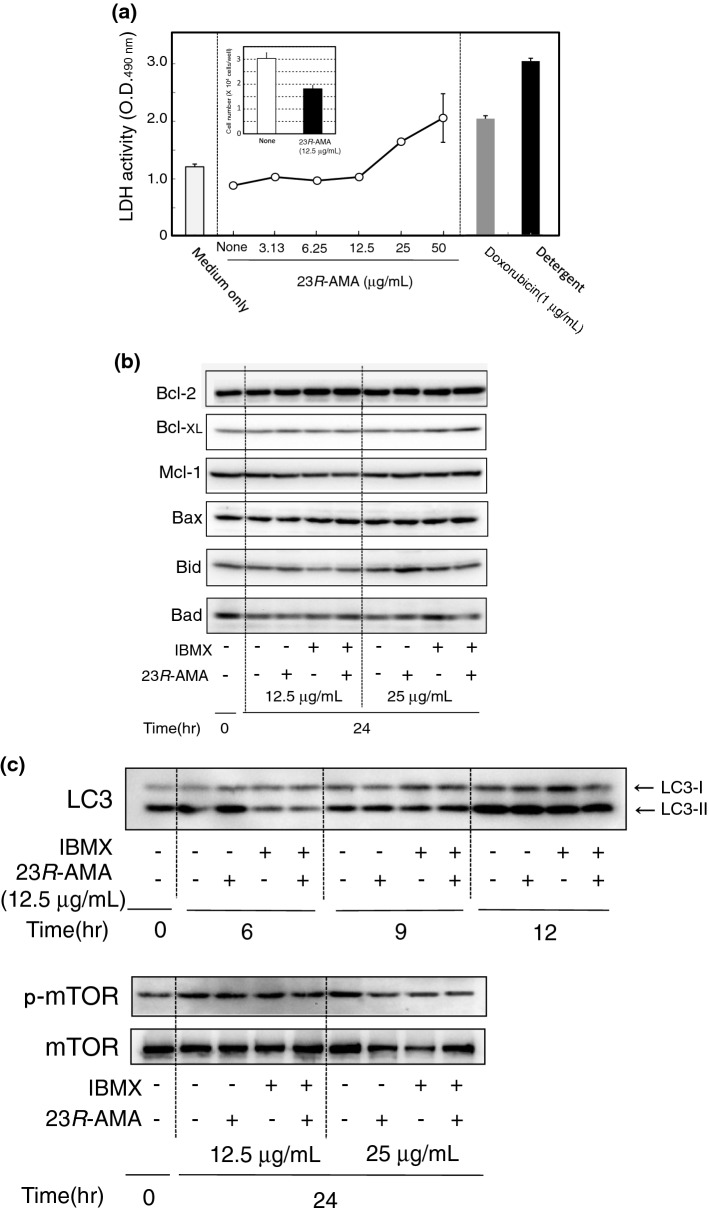
Inhibition of cell proliferation by 23*R*-AMA and influence on apoptosis- and autophagy-related protein expression. **a** B16-F10 cells were seeded in a 96-well plate at a density of 1.0 × 10^4^ cells/well and various concentrations of 23*R*-AMA (3.13–50 μg/mL) were added and cultured for 24 h. LDH activity of the culture supernatant was evaluated by Cytotoxicity LDH Assay Kit-WST. The number of B16-F10 indicated in the insert was counted with a hemocytometer after treatments of 23*R*-AMA (12.5 μg/mL) under the same conditions as the LDH assay. **b**, **c** B16-F10 cells were seeded in a 60-mm dish at 6.0 × 10^5^ cells/dish, and then IBMX (100 μM) was added as a melanin inducer. IBMX-induced cells with/without 23*R*-AMA (12.5, 25 μg/mL) were harvested after 24 h cultivation and WB was performed using specific antibodies to** b** Bcl-2, Bcl-xl, Mcl-1, Bax, Bid, and Bad;** c **LC3, mTOR, and p-mTOR

23*R*-AMA suppressed the production of melanin and reduced the total amount of protein (Fig. 1c). The LDH assay was performed to evaluate the cytotoxic action of 23*R*-AMA. As a result, high dose 23*R*-AMA (50 μg/mL) increased LDH activity to the same degree as doxorubicin (positive control, 1 μg/mL), which indicates cytotoxic effects. Intermediate concentration (25 μg/mL) of 23*R*-AMA also slightly elevated LDH activity, but not from 3.13 to 12.5 μg/mL. 23*R*-AMA (12.5 μg/mL) induced the morphological change of B16-F10 the same as in Fig. 1b (data not shown), and the cell number in a well was suppressed to 60% compared to the non-treated control group (insert in Fig. 2a).

Furthermore, protein expression related to apoptosis and autophagy was investigated by WB. The change in expression of Bcl-2, Bcl-XL, and Mcl-1 preventing apoptosis was not observed in 23*R*-AMA-treated cells by WB. The expression of Bax, Bid, and Bad, which are factors promoting apoptosis, was also not altered by the addition of 23*R*-AMA (Fig. 2b). Furthermore, 23*R*-AMA did not influence LC3-II expression and mTOR phosphorylation, which are autophagy indicators (Fig. 2c).

Therefore, the total protein reduction elicited by 23*R*-AMA is not the consequence of cell death, but it was related to its growth inhibition activity.

## Suppression of cell migration in B16-F10 cells by 23*R*-AMA

**Fig. 3:**
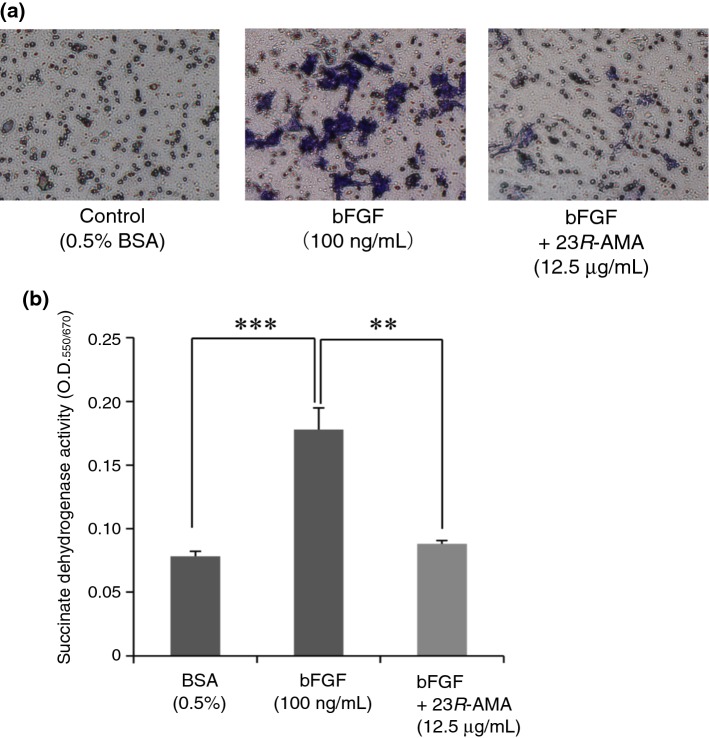
Suppression of cell migration in B16-F10 cells by 23*R*-AMA. Cell culture inserts were placed in a 24-well plate, B16-F10 cells were seeded at a density of 2.0 × 10^4^ cells/mL in the upper layer, and 23*R*-AMA (12.5 μg/mL) was added and cultured for 12 h. After cultivation, the medium of the lower layer was replaced with a medium containing bFGF (100 ng/mL) and incubated for 24 h. **a** B16-F10 cells that migrated to the back side of the membrane induced by bFGF were observed under a microscope after staining with Giemsa solution. **b** After incubation, all non-migratory cells remaining on the upper membrane were removed with a cotton swab and only the migrating cells remaining under the membrane were detected by MTT assay. (**p < 0.01, ***p < 0.005, *n* = 6)

Considering the possibility that the morphological change of B16-F10 induced by 23*R*-AMA affects adhesion, invasion, and metastasis of cancer cells, the effect of 23*R*-AMA on cell migration was investigated. The bFGF-dependent cell migration was inhibited by 23*R*-AMA according to microscopic observations after Giemsa staining (Fig. 3a). In the MTT assay to quantitatively measure the migrating cells, 23*R*-AMA (12.5 μg/mL) significantly suppressed bFGF-induced cell migration to a level comparable to that of the non-induced control group (Fig. 3b).

### 23*R*-AMA-induced phosphorylation of β-catenin and its inhibition of intranuclear accumulation

The expression of MITF protein was suppressed by addition of 23*R*-AMA (Fig. 1d). Since the expression of MITF is known to be regulated by CREB and β-catenin [15, 16], CREB protein expression and its phosphorylation after 23*R*-AMA treatment were analyzed by WB. As a result, no change was evident in the phosphorylation of CREB (data not shown).

**Fig. 4:**
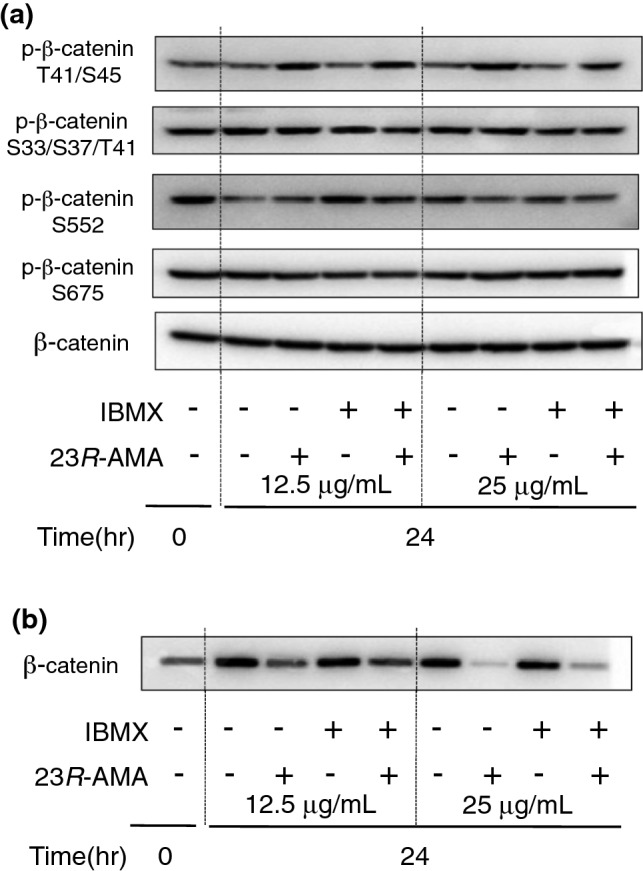
23*R*-AMA-induced phosphorylation of β-catenin and its inhibition of intranuclear accumulation. **a**, **b** B16-F10 cells were seeded in a 60-mm dish at 6.0 × 10^5^ cells/dish and IBMX, as a melanin inducer, and 23*R*-AMA (12.5, 25 μg/mL) were added. Cells were harvested 24 h after the addition of the samples and WB was performed using an antibody against **a** β-catenin, p-β-catenin (T41/S45, S33/S37/T41, S552, S675). **b** B16-F10 cells were treated with 23*R*-AMA under the same conditions as in **a**. Nuclear protein was extracted 24 h after sample addition and WB was performed using an antibody against β-catenin

The activity of β-catenin as a transcription factor is adjusted according to the site to be phosphorylated. Phosphorylation in β-catenin ser45/thr41 at 24 h after 23*R*-AMA addition was enhanced compared with the control in the presence or absence of IBMX, while whole β-catenin content was not altered. On the other hand, phosphorylation at ser33/ser37/thr41, ser552, and ser675 was not affected by 23*R*-AMA treatments (Fig. 4a). Since phosphorylation in ser45/thr41 in β-catenin was reported to suppress its nuclear accumulation [16], β-catenin content in nuclear protein extracts was studied using WB in the same conditions. As a result, the content of β-catenin in the nucleus was obviously suppressed at 24 h from the addition of 23*R*-AMA (Fig. 4b, 12.5 and 25 μg/mL).

## Inhibition of β-catenin downstream gene expression by 23*R*-AMA treatments

**Fig. 5:**
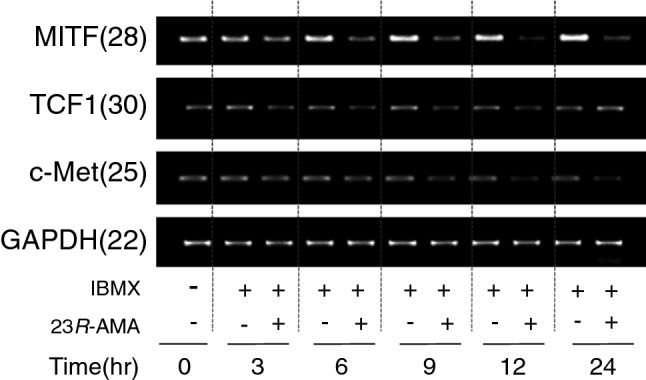
Inhibition of β-catenin downstream gene expression after 23*R*-AMA treatments. B16-F10 cells were seeded in a 60-mm dish at 6.0 × 10^5^ cells/dish and IBMX (100 μM), as a melanin inducer, and 23*R*-AMA (12.5 μg/mL) were added. Total RNA was collected over time from sample addition and cDNA was prepared by RT reaction. RT-PCR was performed using cDNAs, and specific primers of MITF, TCF1, c-Met, and GAPDH, and the expression level of mRNA was semi-quantitatively examined. The numbers in parentheses indicate the number of PCR cycles

Since the suppression of MITF expression is suggested to be caused by 23*R*-AMA-induced suppression of β-catenin accumulation in the nucleus, the change in downstream gene regulated by β-catenin [17] was examined by RT-PCR. As a result, mRNA expression of MITF, TCF1, and c-Met was respectively reduced from 3 to 12 h after the addition of 23*R*-AMA (Fig. 5).

## Inhibition of phosphorylation of c-Raf, MEK, and ERK by 23*R*-AMA treatments

**Fig. 6:**
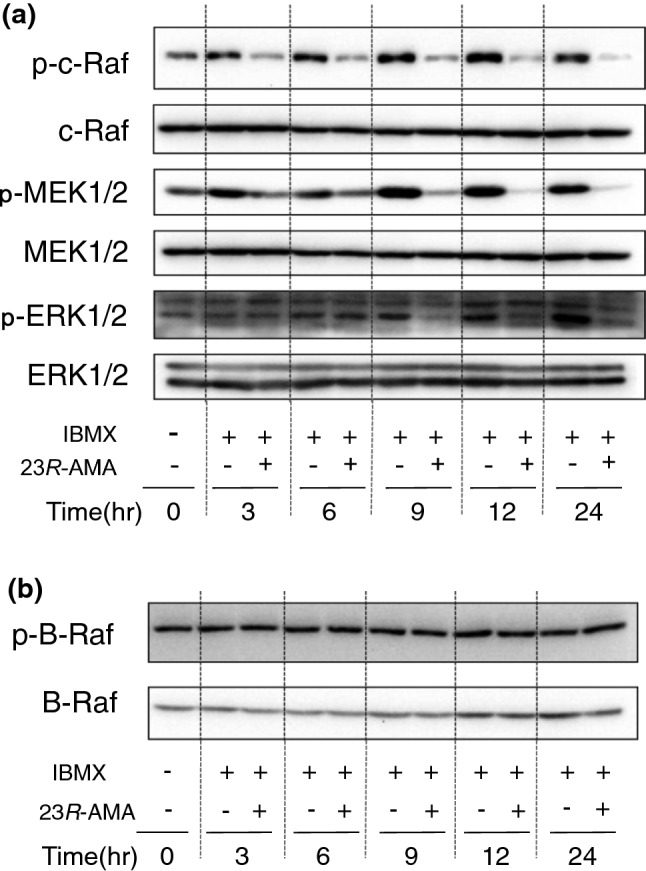
Inhibition of phosphorylation of c-Raf, MEK, and ERK by 23*R*-AMA treatment. B16-F10 cells were seeded in a 60-mm dish at 6.0 × 10^5^ cells/dish, and then IBMX (100 μM) was added as a melanin inducer. IBMX-induced cells with/without 23*R*-AMA (25 μg/mL) were harvested over time from sample addition and WB was performed using specific antibodies to **a** c-Raf, p-c-Raf, MEK1/2, p-MEK1/2, ERK1/2, and p-ERK1/2; **b** B-Raf and p-B-Raf

Phosphorylation of the factors that regulate transcriptional activity of MITF was examined after 23*R*-AMA treatment by WB. Activated ERK is reported to phosphorylate MITF and stimulate its activity [18]. As a result, 23*R*-AMA suppressed phosphorylation of c-Raf, MEK1/2, and ERK1/2 (Fig. 6a). The same analysis was carried out for B-Raf, which is a c-Raf isoform and reported to be frequently activated in malignant melanoma [19, 20], but 23*R*-AMA did not affect B-Raf phosphorylation (Fig. 6b).

## Inhibition of phosphorylation in c-Src/Fyn and FAK by 23*R*-AMA

**Fig. 7:**
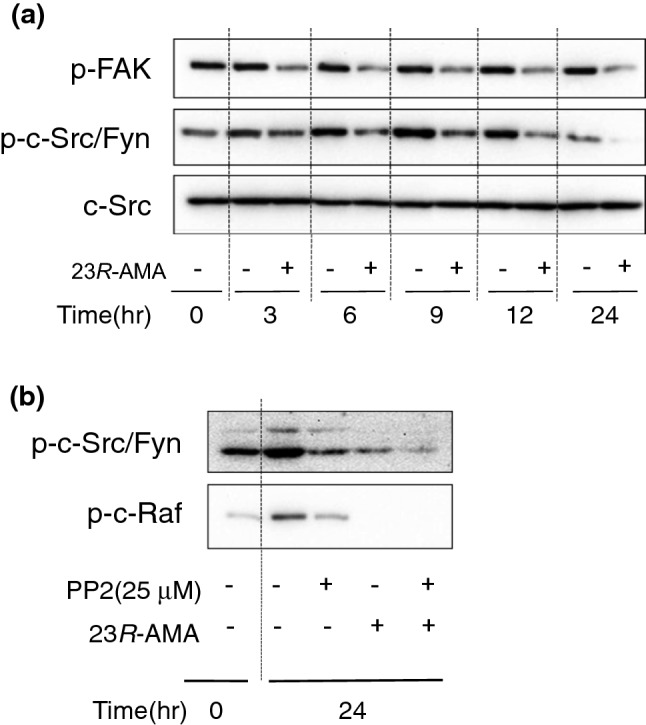
Inhibition of phosphorylation in c-Src/Fyn and FAK by 23*R*-AMA. B16-F10 cells were seeded in a 60-mm dish at 6.0 × 10^5^ cells/dish, and 23*R*-AMA (25 μg/mL) was added. Cells were collected over time 24 h, **a** after sample addition, and WB was performed using specific antibodies against p-FAK, pc-Src/Fyn, or c-Src. **b** B16-F10 cells were seeded in a 60-mm dish at 6.0 × 10^5^ cells/dish, and 23*R*-AMA and PP2 (25 μM) as Src family kinase inhibitor were added. Cells were collected over time 24 h after sample addition and WB was performed using specific antibodies against p-c-Src/Fyn and p-c-Raf

c-Src/Fyn and FAK, which regulate the phosphorylation of c-Raf via Ras [21, 22], were examined as the upstream factors of the c-Raf–MEK1–ERK–MITF signaling axis. Consequently, 23*R*-AMA continuously suppressed c-Src/Fyn and FAK phosphorylation at 3–24 h from the addition (Fig. 7a). Subsequently, in order to confirm whether c-Src/Fyn phosphorylates c-Raf in B16-F10, phosphorylation of c-Raf was examined using PP2 as c-Src family inhibitor. As a result, phosphorylation of c-Raf was attenuated by inhibition of upstream Src family kinase with PP2 pretreatment. Therefore, it was suggested that the phosphorylation of c-Raf is regulated by Src family kinases in B16-F10 cells (Fig. 7b).

## Influence of 23*R*-AMA on integrin expression

**Fig. 8:**
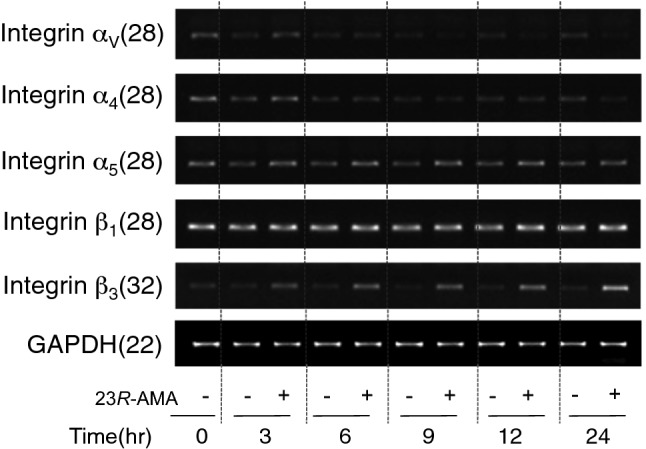
Influence of 23*R*-AMA on integrin expression. B16-F10 cells were seeded in a 60-mm dish at 6.0 × 10^5^ cells/dish with 23*R*-AMA (12.5 μg/mL). Total RNA was collected over time from sample addition and cDNAs were prepared by RT reaction. Using these as a template, RT-PCR was performed with various specific primers for integrins and the expression level of mRNA was semi-quantitatively examined. The numbers in parentheses indicate the number of PCR cycles

FAK phosphorylation is mainly controlled by an integrin known as cell adhesion factor. Thus, the effects of 23*R*-AMA on the integrin expression were investigated by RT-PCR and flow cytometry. Integrin α_V_, α_4_, α_5_, β_1_, and β_3_, which is reported to be expressed in B16-F10 cells was examined [23, 24]. After 23*R*-AMA treatment, the gene expression of integrin α_V_ is reduced at 9–24 h, but that of β_3_ was increased at 3–24 h (Fig. 8). Significant changes in expression of integrin α_4_, α_5_, and β_1_ were not observed. From these results, since 23*R*-AMA was expected to have some effects on the expression of integrins, the expression of integrins on the cell surface was examined by flow cytometry. However, contrary to the results in gene expression, the major integrins expressed on the cell surface in B16-F10 were not influenced by 23*R*-AMA (data not shown).

## Discussion

We isolated 23*R*-AMA, a cycloartane triterpenoid, from a methanol extract of *Garcinia* sp. bark by activity-guided separation [6]. 23*R*-AMA completely suppressed α-MSH/IBMX-induced intracellular melanin accumulation in the B16-F10 melanoma cell line at concentrations over 12.5 μg/mL (Fig. 1b); 23*R*-AMA was more potent than arbutin used as positive control (Fig. 1c). In examining the mechanism of action, protein expression of TYR and MITF, which plays a central role in melanin production, was suppressed by 23*R*-AMA treatments (Fig. 1d). Inhibition of expression of both proteins was also observed in B16-F10 without IBMX. Since many whitening cosmetics, such as arbutin, targeted TYR, 23*R*-AMA was thought to possibly be a candidate for a new whitening agent. However, 23*R*-AMA also induced morphological change and reduction of total protein content in B16-F10 (Fig. 1b, c), features making them unsuitable as cosmetics.

Since recent studies reported that MITF is a crucial target in melanoma therapy [3–5], this study focused on the antitumor activity of 23*R*-AMA. Many cytotoxic effects of current anticancer drugs have been explained by the induction of apoptosis and cell death accompanied by autophagy. However, 23*R*-AMA-induced growth inhibition seemed to be different from apoptotic images generally seen in cell death. Therefore, apoptosis- and autophagy-related factors were investigated after 23*R*-AMA treatment by WB. Significant change of apoptosis-related factor, Bcl-2 family protein, and indicators of autophagy such as LC3-II and mTOR was not observed. These results suggested that total protein reduction by 23*R*-AMA is related to inhibition of proliferation and is not caused by induction of apoptotic cell death and autophagy.

Many compounds with a cycloartane skeleton have been reported to show cytotoxic action, the effects of which are due to induction of apoptosis [25–27]. Previous studies have reported cycloartane triterpenoids having cellular proliferation inhibitory action on cancer cells by affecting cell adhesion and migration [26, 27]. Therefore, the effect of 23*R*-AMA on cell migration, which is involved in invasion and metastasis of cancer cells including melanoma, was investigated. Consequently, 23*R*-AMA significantly suppressed bFGF-induced cell migration compared to the control group (Fig. 3). Although MITF regulates melanin production as a transcription factor, it is also known to control cell cycle, proliferation, survival, and migration [28, 29]. MITF activity is regulated by phosphorylation, and its expression is mainly controlled by CREB and β-catenin [15]. 23*R*-AMA did not affect phosphorylation of CREB. However, 23*R*-AMA increased phosphorylation of β-catenin at Ser 45/Thr 41 (Fig. 4a) and downregulated accumulation of β-catenin in the nucleus (Fig. 4b).

MITF, c-Met, and TCF1 are transcripted as downstream genes of β-catenin [16, 17], which were inhibited by 23*R*-AMA (Fig. 5). TCF1 binds to β-catenin and acts as a transcription factor, controlling not only the target gene including MITF but also the expression of TCF1 itself [15]. Since 23*R*-AMA suppresses gene expression of TCF1 at 3 h from addition, these results correspond to the inhibition of β-catenin accumulation in the nucleus in Fig. 4b.

We demonstrated that 23*R*-AMA influenced β-catenin and TCF that regulate the expression of MITF. However, the regulation of activity of MITF by phosphorylation is mainly performed by the MAPK/ERK signaling pathway [18]. The MAPK/ERK signaling pathway is functionally enhanced in various cancers including melanoma, and many melanoma therapeutic drugs target these pathways. Therefore, influences of 23*R*-AMA on phosphorylation of c-Raf, MEK1/2, and ERK1/2, which plays a central role in the MAPK/ERK signaling pathway, was examined by WB. The results showed that 23*R*-AMA suppressed phosphorylation of c-Raf, MEK1/2, and ERK1/2 (Fig. 6a). On the other hand, B-Raf, an isoform of c-Raf, was examined under the same conditions as c-Raf, but the effect on B-Raf phosphorylation was not observed (Fig. 6b). From these results, it was suggested that 23*R*-AMA inhibits the signaling axis from c-Raf to ERK, and suppressed MITF activation.

Phosphorylation of c-Raf is known to be regulated by Ras, c-Src, and others [30]. Subsequent analysis revealed that 23*R*-AMA suppressed phosphorylation of c-Src/Fyn (Fig. 7a). In addition, 23*R*-AMA suppressed phosphorylation of focal adhesion kinase (FAK) controlling the phosphorylation of c-Src/Fyn (Fig. 7a). Pretreatment of Src family kinase inhibitor PP2, which elucidated the relationship between c-Raf and Src family kinase, inhibited not only phosphorylation of c-Src/Fyn but also that of c-Raf (Fig. 7b). This result suggested that c-Src/Fyn is located upstream of c-Raf and was speculated to control c-Raf phosphorylation via Ras in B16-F10 cells. Taken together, 23*R*-AMA was thought to suppress the c-Raf–MEK-1–ERK signaling axis via inhibition of phosphorylation of FAK-c-Src/Fyn.

In about 40–60% of melanoma, a mutation in B-Raf, 90% of which is a V600E mutation (valine (V) is substituted with glutamic acid (E) at the 600th amino acid), was reported [20, 31]. Currently, a therapeutic agent targeting the V600E mutation has been studied and developed. Drug resistance is reported to occur while its antitumor effect is high at the initial administration [32]. Various research on resistance formation has been conducted, but the detailed mechanism has yet to be elucidated. The most promising theory about the mechanism for reactivation is transactivation of c-Raf [32]. In melanoma cells, B-Raf is responsible for much of the signaling of the MAPK/ERK signaling pathway, and inhibition of B-Raf is compensated for by c-Raf activation and downstream MEK and ERK [21, 30]. Considering such reports, the effect of 23*R*-AMA that suppresses only the activity of c-Raf without affecting B-Raf activity seems to be interesting and significant.

FAK and c-Src/Fyn are defined as mediators of the MAPK/ERK signaling pathway [33, 34], but they are also deeply involved in cell adhesion and migration. In particular, FAK plays a central role in signal transduction mediated by integrins [34]. We preliminarily examined gene expression of integrin α_V_, α_4_, α_5_, β_1_, and β_3_, which are reported to be expressed in B16-F10, and confirmed their expression (Fig. 8). The expression of integrin α_4_, α_5_, and β_1_ was not altered by the addition of 23*R*-AMA. The expression of integrin α_V_ after addition of 23*R*-AMA was downregulated, but β_3_ was upregulated (Fig. 8). Integrin forms a heterodimer on the cell surface and it has been reported that expression of integrin α_V_β_3_, which is a vitronectin receptor, is enhanced in melanoma and contributes largely to cell adhesion, migration, and invasion [35]. However, contrary to the results in Fig. 8, flow cytometric analysis revealed that 23*R*-AMA did not affect the expression of major integrins on the cell surface including α_V_ and β_3_ in B16-F10 (data not shown). Reasons for this discrepancy in results are unknown at present and should be elucidated in the future.

When cells bind to extracellular matrix via integrin, phosphorylation of protein involved in cell adhesion such as FAK, paxillin, and talin, is observed, and Src family kinase subsequently is phosphorylated. These phosphorylations transmit the signals to the formation of cell adhesion plaques and the Rho family that is the main regulator of cell morphology, and cell proliferation and movement with cytoskeletal reconstruction are regulated. Previous studies reported that proteins associated with cell adhesion were not phosphorylated in the cells lacking FAK or Src family kinase, and cell expansion and mobility are markedly reduced [36]. Saracatinib, an inhibitor of c-Src, is a clinically used medicine which has been reported to inhibit cell migration and invasion of melanoma cells without inhibitory effects on proliferation [37]. These reports suggest that 23*R*-AMA-suppressed bFGF-dependent cell migration observed in Fig. 4 might be a consequence of inhibitory effects on FAK and c-Src phosphorylation.

**Fig. 9:**
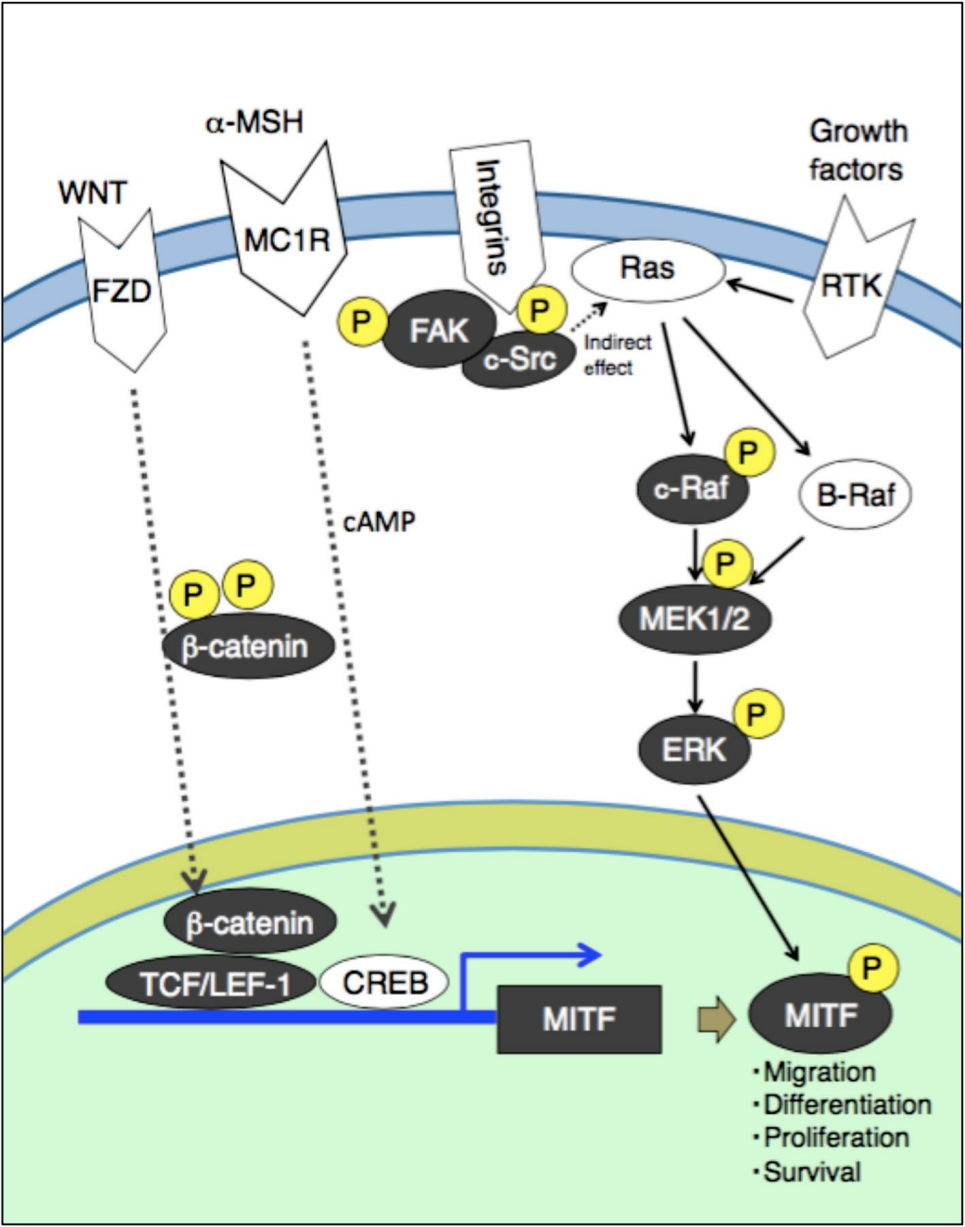
Schematic diagram of the action of 23*R*-AMA in B16-F10 melanoma. Proteins/genes painted black were factors in which downregulation was observed by the addition of 23*R*-AMA. FZD frizzled, MC1R melanocortin 1 receptor, RTKs receptor tyrosine kinases, LEF-1 lymphoid enhancer-binding factor 1

In conclusion, it is inferred that 23*R*-AMA inhibited growth and migration of B16-F10 melanoma by regulating both MITF expression and its activity via regulation of both β-catenin accumulation in the nucleus and the signaling pathway from FAK to ERK (Fig. 9). What should be emphasized in this study is that there has been no report that compounds with a cycloartane skeleton regulate β-catenin and the c-Raf–MEK1–ERK signaling axis including FAK and c-Src. Although we could not identify the target molecule of 23*R*-AMA in this study, if further examination reveals the details of 23*R*-AMA, its effective use can be expected to contribute to future melanoma research.

## Electronic supplementary material

Below is the link to the electronic supplementary material.

Supplementary material 1 (PDF 336 kb)
