# Correction to: Cycloartane triterpenoid (23*R*, 24*E*)-23-acetoxymangiferonic acid inhibited proliferation and migration in B16-F10 melanoma via MITF downregulation caused by inhibition of both β-catenin and c-Raf–MEK1–ERK signaling axis

**DOI:** 10.1007/s11418-020-01401-0

**Published:** 2020-04-10

**Authors:** Toshio Kaneda, Misaki Matsumoto, Yayoi Sotozono, Satoshi Fukami, Alfarius Eko Nugroho, Yusuke Hirasawa, Hadi A. Hamid A, Hiroshi Morita

**Affiliations:** 1 Faculty of Pharmaceutical Sciences, Hoshi University, Ebara 2-4-41 Shinagawa-ku, Tokyo, 142-8501 Japan; 2 Department of Chemistry, Faculty of Science, University Malaya, 50603 Kuala Lumpur, Malaysia

## Correction to: Journal of Natural Medicines (2019) 73:47–58 10.1007/s11418-018-1233-7

The article Cycloartane triterpenoid (23*R*, 24*E*)-23-acetoxymangiferonic acid inhibited proliferation and migration in B16-F10 melanoma via MITF downregulation caused by inhibition of both β-catenin and c-Raf–MEK1–ERK signaling axis, written by Toshio Kaneda, Misaki Matsumoto, Yayoi Sotozono, Satoshi Fukami, Alfarius Eko Nugroho, Yusuke Hirasawa, Hadi A. Hamid A and Hiroshi Morita, was originally published Online First without Open Access. After publication in volume 73 issue 1, page 47–58 the author decided to opt for Open Choice and to make the article an Open Access publication. Therefore, the copyright of the article has been changed to © The Author(s) 2020 and the article is forthwith distributed under the terms of the Creative Commons Attribution 4.0 International License (http://creativecommons.org/licenses/by/4.0/), which permits use, duplication, adaptation, distribution and reproduction in any medium or format, as long as you give appropriate credit to the original author(s) and the source, provide a link to the Creative Commons license, and indicate if changes were made.

The original article was updated.

