# Supplementary material for: Cycloartane triterpenoid (23R, 24E)-23-acetoxymangiferonic acid inhibited proliferation and migration in B16-F10 melanoma via MITF downregulation caused by inhibition of both β-catenin and c-Raf–MEK1–ERK signaling axis
Source: J Nat Med. 2018 Aug 6;73(1):47–58. doi: 10.1007/s11418-018-1233-7 (PMC7188735; doi:10.1007/s11418-018-1233-7)
Supplement: Supplementary file 1 — Supplementary material 1 (PDF 336 kb) [file 11418_2018_1233_MOESM1_ESM.pdf]

NPC Natural Product Communications

2018
Vol. 13
No. 0
1 - 4

Cycloartane Triterpenoids with Anti-melanin Deposition Activity

Alfarius Eko Nugroho[a], Misaki Matsumoto[a], Yayoi Sotozono[a], Toshio Kaneda[a], A. Hamid A. Hadi[b] and Hiroshi Morita[a,*]

[a]*Faculty of Pharmaceutical Sciences, Hoshi University, Ebara 2-4-41 Shinagawa-ku, Tokyo 142-8501, Japan*
[b]*Department of Chemistry, Faculty of Science, University Malaya, 50603 Kuala Lumpur, Malaysia*

moritah@hoshi.ac.jp

Received: February 28th, 2018; Accepted: April 16th, 2018

Bioactivity guided separation of the extract of Malaysian *Garcinia* sp. resulted in the isolation of 23-acetoxy-mangiferonic acid (**1**) and the absolute configuration of C-23 was elucidated to be *R* by the advanced Mosher's method. The anti-melanin deposition activity of **1** was caused by the down regulation of tyrosinase gene expression.

Keywords*:* *Garcinia*, Triterpenoids, Cycloartane, Anti-melanin deposition activity.

Garcinia, a highly diverse pantropical genus consisting of about 300 species, is one of the biggest genera of the Clusiaceae family [1]. Many of the species of *Garcinia* produce edible fruits, with *Garcinia mangostana* (mangosteen) being one of the most well-known species among them. In addition to their edible fruits, the plants of *Garcinia* are also valuable as sources of medicines, color pigments, resins and lumber [2, 3]. Phytochemically, this genus is mostly known for its unique prenylated xanthones and benzophenones [2, 4-9], though triterpenoids [10, 11] and flavonoids [12-14] were also reported.

In our search for new bioactive compounds [15-31], we screened Malaysian plants extracts for anti-melanin deposition activity. The bark MeOH extract of *Garcinia sp.* showed a dose-dependent inhibition of melanin deposition activity from 12.5 μg/mL. The extract was then partitioned into *n*-hexane, EtOAc, *n*-BuOH and water fractions for bioassay-guided isolation purpose, and the *n*-hexane and EtOAc fractions showed promising activity. Activity-guided separation of both active fractions, led to the isolation of compound **1** (Figure 1) as the active compound (IC_{50} 9.9 μg/mL = 19.4 μM).

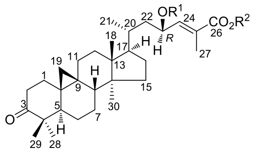

1: R^1 = Ac; R^2 = H
2: R^1 = H; R^2 = H
3: R^1 = H; R^2 = Me

Figure 1: Structures of **1 - 3**.

Based on 1D and 2D NMR data, compound **1** was deduced to be 23-acetoxy-mangiferonic acid. 23-acetoxy-mangiferonic acid was previously reported by Anjaneyulu *et al.* as a component of an acetylation product mixture of a mixture of 23-hydroxy-mangiferonic acid and 22-hydroxy-isomangiferolic acid [32]. Hence this is the first report of the isolation of 23-acetoxy-mangiferonic acid as a pure compound, and the first complete assignment of its ^{1}H and ^{13}C NMR spectra (Table 1). To determine the absolute configuration of C-23, **1** was hydrolyzed to obtain **2**. **2** was then methylated to obtain its methyl ester derivative **3**. The absolute configuration of C-23 in **3** was determined to be *R* by using the

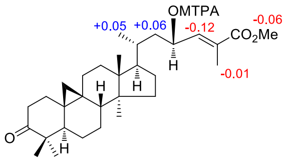

Figure 2: Δδ (δ*S*-δ*R*) values of the MTPA esters of **3**.

Table 1: ^{1}H and ^{13}C NMR data of **1** in $CDCl_3$.

No.	δC	δH (*J*, Hz)	No.	δC	δH (*J*, Hz)
1	33.3	1.51 (1H, m)	16	28.1	1.28 (1H, m)
		1.82 (1H, td, 13.3, 3.7)			1.90 (1H, m)
2	37.3	2.28 (1H, br.d, 14.0)	17	52.5	1.54 (1H, m)
		2.68 (1H, td, 14.0, 6.5)	18	17.9	0.97 (3H, s)
3	216.7		19	29.4	0.55 (1H, d, 4.2)
4	50.1				0.75 (1H, d, 4.2)
5	48.3	1.68 (1H, dd, 12.0, 4.1)	20	32.5	1.48 (1H, m)
6	21.4	0.92 (1H, m)	21	18.2	0.92 (3H, d, 6.0)
		1.54 (1H, m)	22	40.1	1.08 (1H, m)
7	25.7	1.10 (1H, m)			1.90 (1H, m)
		1.32 (1H, m)	23	68.9	5.60 (1H, td, 8.4, 2.6)
8	47.7	1.55 (1H, m)	24	141.9	6.65 (1H, dd, 8.4, 1.1)
9	21		25	128.2	
10	25.9		26	172.5	
11	26.5	1.16 (1H, m)	27	12.4	1.89 (3H, br.s)
		2.01 (1H, m)	28	22.1	1.00 (3H, s)
12	32.7	1.62 (2H, m)	29	20.7	1.07 (3H, s)
13	45.4		30	19.1	0.86 (3H, s)
14	48.7		COMe	170.5	
15	35.4	1.29 (2H, m)	COMe	20.9	2.04 (3H, s)

advanced Mosher's method (Figure 2). Hence, the absolute configuration of C-23 in **1** and **2** was deduced to be *R*. Thus, compound **1** was deduced to be (23*R*, 24*E*)-23-acetoxy-mangiferonic acid and compound **2** was deduced to be (23*R*, 24*E*)-23-hydroxy-mangiferonic acid.

23-hydroxy-mangiferonic acid with unknown C-23 configuration was isolated from *G. cornea* [33]. The ^{1}H and ^{13}C NMR data of **2** were identical to those of 23-hydroxy-mangiferonic acid isolated from *G. cornea*. In addition, it has been reported that the chemical shifts of C-22 to C-25, C-26 and C-28 of two C-23-epimers of 23-hydroxy-mangiferolic acid were different [32]. Thus, the 23-

hydroxy-mangiferonic acid isolated from *G. cornea* should also have 23*R* configuration.

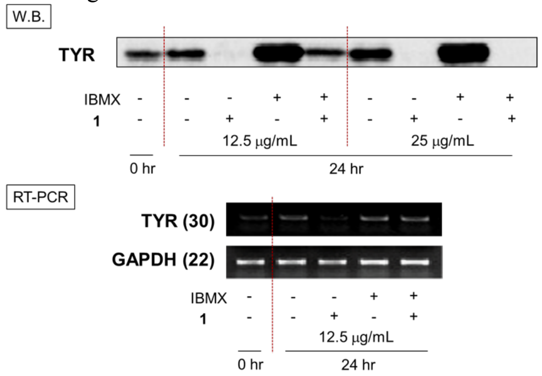

Figure 3: Inhibitory effect of **1** on tyrosinase protein and gene expression. Glyceraldehyde 3-phosphate dehydrogenase (GAPDH) was used as a control. Numbers within parentheses indicate the number of PCR cycles.

To examine the action of **1**, tyrosinase protein expression was analyzed by western blotting. Tyrosinase protein expression was induced by treatment with IBMX. As shown in Figure 2, addition of 12.5 or 25 μg/mL of **1** potently inhibited the tyrosinase protein expression at 24 hours. Furthermore, **1** inhibited the tyrosinase protein expression even without the addition of IBMX. Next, tyrosinase gene expression was analyzed by reverse-transcription polymerase chain reaction (RT-PCR). As shown in Figure 3, tyrosinase mRNA expression was elevated after 24 hours of the induction, and was suppressed by the addition of **1** (12.5 μg/mL). These results suggest that the anti-melanin deposition activity of **1** was caused by the down regulation of tyrosinase gene expression.

Experimental

General experimental procedures: Optical rotations were measured on a JASCO DIP-1000 polarimeter, UV spectra on a Shimadzu UVmini-1240 spectrophotometer, and IR spectra on a JASCO FT/IR-4100 spectrophotometer. High-resolution ESI MS were obtained on a LTQ Orbitrap XL (Thermo Scientific). ^{1}H and 2D NMR spectra were measured on a 400 MHz spectrometer at 300K, and ^{13}C NMR spectra on a 100 MHz spectrometer. Residual solvent chemical shifts were used as internal standard, δ_H 7.26 and δ_C 77.0. Standard pulse sequences were used for the 2D NMR experiments.

Material: The barks of *Garcinia sp.* were collected in Johor, Malaysia in August 2003. The botanical identification was made by Mr. Teo Leong Eng, Faculty of Science, University of Malaya. Voucher specimens (Herbarium No. 5044) are deposited in the Herbarium of Chemistry Department, University of Malaya.

Extraction and isolation: The barks of *Garcina sp.* (5 g) were extracted with methanol to obtain 1.4 g of extract. The methanol extract was successively partitioned with *n*-hexane, ethyl acetate, *n*-butanol and water, and the hexane-soluble materials (63.0 mg) were further separated with a silica gel column (*n*-hexane/EtOAc, 1:0 → 1:1) to obtain (23*R*, 24*E*)-23-acetoxymangiferonic acid (**1**, 19.4 mg). Separation of the ethyl acetate fraction (295 mg) by silica gel column ($CHCl_3$/MeOH 1:0 → 9:1) and followed by ODS columns (MeOH/H_2O, 4:1) yielded additional **1** (156 mg). The total yield of **1** is 3.51%

(23R, 24E)-23-acetoxy-mangiferonic acid (1)
white amorphous solid.
$[\alpha]_D^{17}$: 12.8 (*c* 1.0, $CHCl_3$).
IR (KBr) ν_{max}: 3400, 1710 and 1695 cm^{-1}.
UV/Vis λ_{max} (MeOH) nm (log ε): 211 (4.27) nm.
CD (MeOH) λ_{max} ($\Delta\varepsilon$): 208 (4.03), 234 (-1.90) and 296 (-0.89) nm.
^{1}H and ^{13}C NMR: Table 1
MS (ESI): *m/z* 535 [M+Na]$^+$;
HRESITOFMS: *m/z* ([M+Na]$^+$ calcd for $C_{32}H_{48}O_5Na$: 535.33994; found 535.34206

Alkaline hydrolysis and methylation of 1: To a solution of **1** (0.8 mg in 100 μL MeOH), 100 μL of 2M $NaOH_{(aq)}$ was added, and the resulting mixture was left overnight at r.t. The reaction mixture was then neutralized with 2M $HCl_{(aq)}$ and partitioned with $CHCl_3$. The $CHCl_3$ fraction was dried under N_2 blow and the resulting residue (23-hydroxy-mangiferonic acid, **2**, 0.7 mg) was then dissolved in 50 μL MeOH. To the MeOH solution, 20 μL of TMS-diazomethane (10% in *n*-hexane) was added and was left at r.t. After 10 min., the reaction mixture was dried under N_2 blow and the resulting residue was subjected to SiO_2 column chromatography ($CHCl_3$) to obtain methyl 23-hydroxy-mangiferonate (**3**, 0.8 mg).

Modified Mosher's method of 3: First, (*R*)-α-methoxy-α-(trifluoromethyl)phenylacetic (MTPA) chloride (2 μL) was added to a solution of **3** (0.4 mg) with a catalytic amount of 4-(dimethylamino)pyridine and 1 μL of triethylamine in 50 μL of CH_2Cl_2, and the solution was allowed to stand at room temperature overnight. The residue obtained under N_2 blow was subjected to SiO_2 column chromatography ($CHCl_3$) to obtain the (*S*)-MTPA ester of **3** (**4**). The same procedure was used to obtain of the (*R*)-MTPA ester of **3** (**5**).

(*S*)-MTPA ester of **3** (**4**)
^{1}H NMR (400 MHz, $CDCl_3$): 0.98 (3H, d, *J* = 6.0 Hz, H-21), 1.98 (1H, m, H-22), 2.04 (1H, m, H-22), 6.52 (1H, d, *J* = 8.4 Hz, H-24), 2.00 (3H, s, H-27), 3.73 (3H, s, OMe),
MS (ESI): *m/z* 723 [M+Na]$^+$.

(*R*)-MTPA ester of **3** (**5**)
^{1}H NMR (400 MHz, $CDCl_3$): 0.93 (3H, d, *J* = 6.0 Hz, H-21), 1.92 (1H, m, H-22), 1.98 (1H, m, H-22), 6.64 (1H, d, *J* = 8.4 Hz, H-24), 2.01 (3H, s, H-27), 3.79 (3H, s, OMe),
MS (ESI): *m/z* 723 [M+Na]$^+$.

Anti-melanin deposition activity and cytotoxicity: Cells were seeded on a 24 well plate at 1.0×10^5 cells/mL, and treated with 100 μM 3-Isobutyl-1-methylxanthine (IBMX, Wako, Osaka, Japan), 0.25 μM of α-melanocyte stimulating hormone (α-MSH, Sigma, St. Louis, MO, USA) and the samples for 72 hours. After this treatment, to quantify the melanin deposition, the cells were lysed by 1N NaOH at 95°C, and the absorbance at 360 nm was measured. In addition, the samples cytotoxicity was evaluated *via* measurement of the total protein contents using Coomassie Protein Assay Reagent (Thermo scientific, Rockford, IL, USA), and measurement of the absorbance at 595 nm. 750 μM of Arbutin (Sigma, St. Louis, MO, USA) was used as positive control.

Western blot: B16-F10 cells were seeded on a 6 cm dish at 6.0×10^5 cells/dish, and treated with IBMX (100 μM) and **1** (12.5 and 25 μg/mL) for 24 hours. After this treatment, cells were washed with PBS, and lysed in 100 μL of 2×Laemmli buffer [125 mM Tris-HCl (pH 6.8)、4% SDS、20% glycerol、0.0025% bromophenol blue(BPB)]. Cell lysates were sonicated to shear DNA. Protein levels were determined subsequently *via* BCA assay (ThermoScientific, Rockford, IL, USA). After separation in 7.5% SDS-PAGE, protein was transferred onto a polyvinylidene

difluoride (PVDF) membrane (Amersham Hybond^{TM}-P, GE Healthcare, Buckinghamshire, UK). The membrane was blocked with 5% skim milk in TBS/T for 1 hour and then incubated with anti-tyrosinase antibody (Santa Cruz Biotechnology, CA, USA) overnight at 4°C. For protein detection, the membranes were incubated with donkey anti-goat IgG-HRP-conjugated secondary antibody (Santa Cruz Biotechnology).

Reverse transcription-PCR: B16-F10 cells were seeded on a 6 cm dish at 6.0×10^5 cells/dish, and treated with IBMX (100 μM) and **1** (12.5 μg/mL) for 24 hours. After this treatment, cells were washed with PBS, and lysed in 1 mL of Trizol reagent (Ambion, Austin, TX, USA). Reverse transcriptions were performed using the ReverTra Ace kit (Toyobo, Osaka, Japan). Polymerase chain reaction (PCR) assays were carried out using the PCR kit from Qiagen (Hilden, Germany). For each PCR reaction, 0.1 μg/μL of cDNA was used. The synthesized forward and reverse primer sequences are as follows: GAPDH (forward: 5'-TCATCATCTCCGCCCCTTC-3', reverse: 5'-TGCCTGCTTCACCACCTTCT-3'), tyrosinase (forward: 5'-GATCAGAAGAGTATAATAGCCAT-3', reverse: 5'-CAATATAAGGGCTGTAAAAGCCT-3'). All PCR products were separated by 1.5% agarose gel electrophoresis and visualized by ethidium bromide staining.

Acknowledgments - This work was supported by a grant from Grants in-Aid for Scientific Research from JSPS.

References

[1] Gustafsson MHG, Bittrich V, Stevens PF. (**2002**) Phylogeny of Clusiaceae Based on rbcL sequences. *International Journal of Plant Sciences*, ***163***, 1045-1054.

[2] Chantarasriwong O, Batova A, Chavasiri W, Theodorakis EA. (**2010**) Chemistry and Biology of the Caged Garcinia Xanthones. *Chemistry – A European Journal*, ***16***, 9944-9962.

[3] Obolskiy D, Pischel I, Siriwatanametanon N, Heinrich M. (**2009**) *Garcinia mangostana* L.: a phytochemical and pharmacological review. *Phytotherapy Research*, ***23***, 1047-1065.

[4] Masullo M, Bassarello C, Bifulco G, Piacente S. (**2010**) Polyisoprenylated benzophenone derivatives from the fruits of *Garcinia cambogia* and their absolute configuration by quantum chemical circular dichroism calculations. *Tetrahedron*, ***66***, 139-145.

[5] Li P, Anandhi Senthilkumar H, Figueroa M, Wu S-B, Fata JE, Kennelly EJ, Long C. (**2016**) UPLC-QTOFMSE-Guided Dereplication of the Endangered Chinese Species *Garcinia paucinervis* to Identify Additional Benzophenone Derivatives. *Journal of Natural Products*, ***79***, 1619-1627.

[6] Ji F, Li Z, Liu G, Niu S, Zhao N, Liu X, Hua H. (**2012**) Xanthones with antiproliferative effects on prostate cancer cells from the stem bark of *Garcinia xanthochymus*. *Natural Product Communications*, ***7***, 53-56.

[7] Chin Y-W, Shin E, Hwang BY, Lee MK. (**2011**) Antifibrotic constituents from *Garcinia mangostana*. *Natural Product Communications*, ***6***, 1267-1268.

[8] Mohd Khalid R, Jabit ML, Abas F, Stanslas J, Shaari K, Lajis NH. (**2007**) Cytotoxic xanthones from the leaves of *Garcinia urophylla*. *Natural Product Communications*, ***2***, 271-276.

[9] Komguem J, Lannang AM, Tangmouo JG, Louh GN, Ngounou FN, Lontsi D, Choudhary MI, Sondengam BL. (**2006**) Polyanxanthone, a xanthone from the stem bark of *Garcinia polyantha*. *Natural Product Communications*, ***1***, 363-365.

[10] Nguyen HD, Trinh BTD, Tran QN, Nguyen HD, Pham HD, Hansen PE, Duus F, Connolly JD, Nguyen L-HD. (**2011**) Friedolanostane, friedocycloartane and benzophenone constituents of the bark and leaves of *Garcinia benthami*. *Phytochemistry*, ***72***, 290-295.

[11] Mori R, Nugroho AE, Hirasawa Y, Wong CP, Kaneda T, Shirota O, Hadi AHA, Morita H. (**2014**) Opaciniols A–C, new terpenoids from *Garcinia opaca*. *Journal of Natural Medicines*, ***68***, 186-191.

[12] Ito T, Yokota R, Watarai T, Mori K, Oyama M, Nagasawa H, Matsuda H, Iinuma M. (**2013**) Isolation of Six Isoprenylated Biflavonoids from the Leaves of *Garcinia subelliptica*. *Chemical and Pharmaceutical Bulletin*, ***61***, 551-558.

[13] Al-Shagdari A, Alarcon AB, Cuesta-Rubio O, Piccinelli AL, Rastrelli L. (**2013**) Biflavonoids, main constituents from *Garcinia bakeriana* leaves. *Natural Product Communications*, ***8***, 1237-1240.

[14] Kaikabo AA, Samuel BB, Eloff JN. (**2009**) Isolation and activity of two antibacterial biflavonoids from leaf extracts of *Garcinia livingstonei* (Clusiaceae). *Natural Product Communications*, ***4***, 1363-1366.

[15] Nugroho AE, Hashimoto A, Wong C-P, Yokoe H, Tsubuki M, Kaneda T, Hadi AHA, Morita H. (**2018**) Ceramicines M–P from *Chisocheton ceramicus*: isolation and structure–activity relationship study. *Journal of Natural Medicines*, ***72***, 64-72.

[16] Nugroho AE, Inoue D, Wong CP, Hirasawa Y, Kaneda T, Shirota O, Hadi AHA, Morita H. (**2018**) Reinereins A and B, new onocerane triterpenoids from *Reinwardtiodendron cinereum*. *Journal of Natural Medicines*, 72, 588-592.

[17] Nugroho AE, Sasaki T, Kaneda T, Hadi AHA, Morita H. (**2017**) Calofolic acids A–F, chromanones from the bark of Calophyllum scriblitifolium with vasorelaxation activity. *Bioorganic & Medicinal Chemistry Letters*, ***27***, 2124–2128.

[18] Iijima C, Wong CP, Nugroho AE, Sotozono Y, Someya S, Hirasawa Y, Kaneda T, Hadi AHA, Morita H. (**2016**) Anti-melanin deposition activity of ceramicines from *Chisocheton ceramicus*. *Journal of Natural Medicines*, ***70***, 702-707.

[19] Nugroho AE, Chin-Piow W, Hirasawa Y, Janar J, Kaneda T, Shirota O, Morita H. (**2016**) Daphnane Diterpenoids from *Daphne altaica*. *Natural Product Communications*, ***11***, 1073-1075.

[20] Wong CP, Seki A, Horiguchi K, Shoji T, Arai T, Nugroho AE, Hirasawa Y, Sato F, Kaneda T, Morita H. (**2015**) Bisleuconothine A Induces Autophagosome Formation by Interfering with AKT-mTOR Signaling Pathway. *Journal of Natural Products*, ***78***, 1656-1662.

[21] Nugroho AE, Sugiura R, Momota T, Hirasawa Y, Wong CP, Kaneda T, Hadi AHA, Morita H. (**2015**) Dysosesquiflorins A and B, sesquiterpenoids from *Dysoxylum densiflorum*. *Journal of Natural Medicines*, ***69***, 411-415.

[22] Morita H, Nugroho AE, Nagakura Y, Hirasawa Y, Yoshida H, Kaneda T, Shirota O, Ismail IS. (**2014**) Chrotacumines G–J, chromone alkaloids from *Dysoxylum acutangulum* with osteoclast differentiation inhibitory activity. *Bioorganic & Medicinal Chemistry Letters*, ***24***, 2437-2439.

[23] Nugroho AE, Momota T, Sugiura R, Hanzawa M, Yajima E, Nagakura Y, Yasuda N, Hirasawa Y, Wong CP, Kaneda T, Hadi AHA, Fukaya H, Morita H. (**2014**) Dysotriflorins A–M, triterpenoids from *Dysoxylum densiflorum*. *Tetrahedron*, ***70***, 9661-9667.

[24] Wong CP, Deguchi J, Nugroho AE, Kaneda T, Hadi AHA, Morita H. (**2013**) Ceramicines from *Chisocheton ceramicus* as lipid-droplets accumulation inhibitors. *Bioorganic & Medicinal Chemistry Letters*, ***23***, 1786-1788.

[25] Nugroho AE, Hirasawa Y, Wong CP, Kaneda T, Hadi AHA, Shirota O, Ekasari W, Widyawaruyanti A, Morita H. (**2012**) Antiplasmodial indole alkaloids from *Leuconotis griffithii*. *Journal of Natural Medicines*, ***66***, 350-353.

[26] Nugroho AE, Sugai M, Hirasawa Y, Hosoya T, Awang K, Hadi AHA, Ekasari W, Widyawaruyanti A, Morita H. (**2011**) New Antiplasmodial Indole Alkaloids from *Hunteria zeylanica*. *Bioorganic & Medicinal Chemistry Letters*, ***21***, 3417-3419.

[27] Nugroho AE, Hirasawa Y, Hosoya T, Awang K, Hadi AHA, Morita H. (**2010**) Bisleucocurine A, a novel bisindole alkaloid from *Leuconotis griffithii*. *Tetrahedron Letters*, ***51***, 2589-2592.

[28] Hirasawa Y, Hara M, Nugroho AE, Sugai M, Zaima K, Kawahara N, Goda Y, Awang K, Hadi AHA, Litaudon M, Morita H. (**2010**) Bisnicalaterines B and C, Atropisomeric Bisindole Alkaloids from *Hunteria zeylanica*, Showing Vasorelaxant Activity. *The Journal of Organic Chemistry*, ***75***, 4218-4223.

[29] Nugroho AE, Hirasawa Y, Kawahara N, Goda Y, Awang K, Hadi AHA, Morita H. (**2009**) Bisnicalaterine A, a Vobasine−Vobasine Bisindole Alkaloid from *Hunteria zeylanica*. *Journal of Natural Products*, ***72***, 1502-1506.

[30] Nugroho AE, Nakamura H, Inoue D, Hirasawa Y, Wong CP, Kaneda T, Hadi AHA, Morita H. (**2018**) Polyisoprenylated acylphloroglucinols from *Garcinia nervosa*. *Natural Product Communications*, ***13***, 367-369.

[31] Nugroho AE, Moue M, Sasaki T, Shirota O, Hadi AHA, Morita H. (**2018**) Yohimbine-related alkaloids from *Tabernaemontana corymbosa*. *Natural Product Communications*, ***13***, 347-350.

[32] Anjaneyulu V, Satyanarayana P, Viswanadham KN, Jyothi VG, Rao KN, Radhika P. (**1999**) Triterpenoids from *Mangifera indica*. *Phytochemistry*, ***50***, 1229-1236.

[33] Elfita E, Muharni M, Latief M, Darwati D, Widiyantoro A, Supriyatna S, Bahti HH, Dachriyanus D, Cos P, Maes L, Foubert K, Apers S, Pieters L. (**2009**) Antiplasmodial and other constituents from four Indonesian *Garcinia* spp. *Phytochemistry*, ***70***, 907-912.
